# Retraction: Novel Role of Phosphorylation-Dependent Interaction between FtsZ and FipA in Mycobacterial Cell Division

**DOI:** 10.1371/journal.pone.0264672

**Published:** 2022-02-24

**Authors:** The PLOS ONE Editors

Following the publication of this article [1], concerns were raised regarding the results presented in several figures. Specifically,

- In Fig. 2A and 9C, the following results appear similar despite being used to represent different experimental conditions:
  ○ The Fig. 2A immunoblotted (IB): FipA panel (lanes 1 and 2) and the Fig. 9C IB: FtsZ panel (bottom right) flipped horizontally.
  ○ The Fig. 2A IB: FtsZ panel and the Fig. 9C IB: anti-S panel.
  ○ The Fig. 9C IB: FipA panel (top right) and the Fig. 9C IB: FtsQ panel (center right).
- In Fig. 2A (top panel), lanes 1 and 2 are labelled IB: FipA and lane 3 is labelled IB: PPK1, but no explanation was provided for how the blot was probed with antibodies against two proteins, or how proteins of different expected molecular weights are presented side-by-side.
- In Fig. 3A (top left panel), there appears to be repetitive elements in the background pattern of lane 1.
- In Fig. 4A, there appears to be vertical discontinuity between lanes 2 and 3 of the Coomassie gel.
- In Fig. 6C, when color levels are adjusted, the overall background is lacking detail, and there are regular shapes around the areas of fluorescence.
- In Fig. 9A IB: FipA panel, there appears to be vertical discontinuity between lanes 1 and 2.
- In Figure S6A, when color levels are adjusted, the background is lacking detail, and areas near the cells are unlike the overall background.

The corresponding author provided raw images for western blots presented in Fig. 2A, 3A and 9C, and the Coomassie gel presented in Fig. 4A. The raw images for western blots do not appear to show the full blot area. The corresponding author stated that raw image data were not available for Fig. 6C, 9A, and Figure S6A. The corresponding author apologized that raw data for all figures could not be retrieved due to the time elapsed since the experiments were performed and due to inadequate archiving.

The corresponding author stated that the duplicate panels in Fig. 2A and 9C were due to errors in the preparation of Fig. 9C, and provided a version of Fig. 9 in which the panels of concern were replaced. For Fig. 4A, the corresponding author noted that lanes from different parts of the Coomassie gel were cropped together for presentation, and this explanation was confirmed by the raw image provided.

The raw image data provided for Fig. 2A (top panel) and Fig. 3A did not satisfactorily resolve the concerns described above. Raw image data was not available for Fig. 9A, and the author provided replicate data. The *PLOS ONE* Editors remain concerned about these figures.

The corresponding author stated that the issue in Fig. 6C could be due to background noise, and they were unable to identify the issue in Figure S6A. As no raw data were available, the concerns in these figures were not resolved.

The issues impact multiple figure panels, and underlying data were available for only some of the affected figures. The raw data or replacement panels that were available did not satisfactorily address all the above issues. In light of the concerns affecting multiple figure panels that question the integrity of these data, the *PLOS ONE* Editors retract this article.

JB and MK agreed with the retraction. KS, TH, PM, PC, and PD either did not respond directly or could not be reached.
